# Chronotherapy based on modified-release hydrocortisone to restore the physiological cortisol diurnal rhythm

**DOI:** 10.1007/s13346-022-01183-w

**Published:** 2022-05-26

**Authors:** Martin J. Whitaker, Hiep Huatan, Richard J. Ross

**Affiliations:** 1 Diurnal Ltd, Cardiff, UK; 2 Department of Oncology and Metabolism, University of Sheffield, Sheffield, UK

**Keywords:** Chronotherapy, Circadian, Cortisol, Hydrocortisone, Efmody, Hormone, Multi-particulate

## Abstract

In this inspirational note, we describe the development of an endocrine chronotherapy to restore the physiological rhythm of the essential adrenal stress hormone, cortisol. The challenges included demonstrating the circadian rhythm of the drug target, creating a drug formulation that replicated that rhythm and then proving benefit in clinical trials. The physiological cortisol circadian rhythm is well defined with cortisol levels high on waking and low on going to sleep. We experimented with different formulation technologies including modified-release tablets and multi-particulates to replicate the cortisol rhythm where absent through disease. We describe the development of Efmody^®^, a modified-release formulation of hydrocortisone, which replicates the cortisol diurnal rhythm and improves the disease control of congenital adrenal hyperplasia, the commonest hereditary form of adrenal insufficiency. This program shows it is possible, through modified-release technology, to treat chronic endocrine diseases with physiological replacement to preserve health for life.

## Introduction to chronotherapy

A central tenet in medicine is that disruption of homeostatic mechanisms leads to disease, and effective therapy must re-establish normal physiology [1]. The sun imposes a 24-h periodicity to life on earth that regulates much of human behaviour. Circadian rhythms have evolved in virtually all organisms to maintain homeostasis through the 24-h day/night cycle. In endocrine disorders such as adrenal insufficiency, replicating the hormonal rhythms through chronotherapy is essential to restore normal physiology and maintain optimal health. The physiology of hormones is more complex than that of non-native drugs. Hormones are secreted with specific but varied rhythms, frequently bound to multiple cognate binding proteins and actively transported and cleared through various enzymatic pathways in multiple organs. To replicate a hormone’s rhythm, it is important to first understand its physiology and then devise a formulation that can replicate that physiology. In this inspirational note, we describe the unmet need for and the clinical development of Efmody^®^, a modified-release formulation of hydrocortisone that replicates the cortisol circadian rhythm. The innovation is in using drug delivery technology to deliver cortisol in a way that replicates human physiology.

## The unmet need for chronotherapy in adrenal insufficiency

**Fig. 1:**
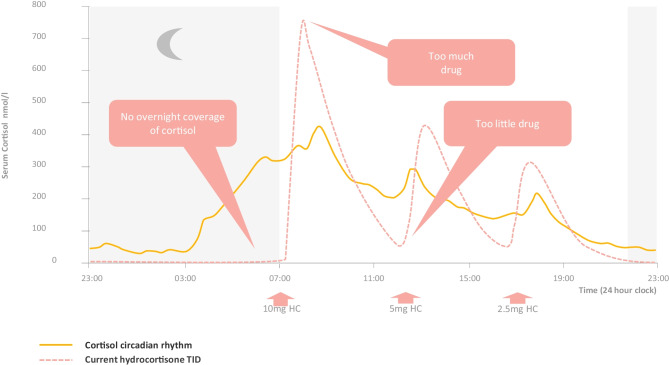
The circadian rhythm of cortisol (yellow) and thrice-daily immediate-release hydrocortisone (HC) replacement (yellow) showing variable pharmacokinetic cortisol replacement during the day time period (ca. 07:00 h to 22:00 h) but with no overnight replacement of cortisol during the nighttime period (ca. 22:00 h to 07:00 h). Adapted from Debono et al. JCEM [6]

Adrenal insufficiency results from a deficiency in the essential glucocorticoid stress hormone cortisol, and patients may die from an adrenal crisis, circulatory collapse, if cortisol is not replaced [2]. Adrenal insufficiency is either primary due adrenal failure (congenital adrenal hyperplasia (CAH) and Addison’s disease), secondary due to pituitary failure or tertiary due to adrenal suppression following anti-inflammatory glucocorticoid therapy. Since the 1960s, patients with adrenal insufficiency have been replaced with hydrocortisone (the synthetic form of the endogenous hormone, cortisol); however, despite replacement, patients still have an increased mortality, up to 5 times that of the healthy population, patients feel tired, and those with CAH have poor disease control [3, 4]. Immediate-release hydrocortisone cannot replace the normal circadian rhythm of cortisol (Fig. 1), particularly during the nighttime period, and patients are frequently exposed to excess or deficient cortisol resulting in poor disease control and the increased mortality which is particularly evident in patients with CAH [5]. Cortisol has a distinct circadian rhythm with low levels on going to sleep; levels start to rise around 02:00–04:00 h, peak on waking and then slowly decline over the day to fall to low levels in the evening (Fig. 1) [6]. Hydrocortisone replacement therapy in adrenal insufficiency is given twice or thrice daily with the first dose given on waking. Hydrocortisone has a relatively short half-life (1.5 h) [7], so patients wake with very low or undetectable levels of cortisol at the time when their cortisol level should be at its highest. This is associated with fatigue, and in the case of CAH, poor disease control than among other clinical outcomes results in poor quality of life as well as cardiovascular, metabolic and reproductive complications, such as infertility. There is therefore a need for chronotherapy to replace the diurnal rhythm of cortisol.

## The rhythm of life: understanding cortisol physiology

**Fig. 2:**
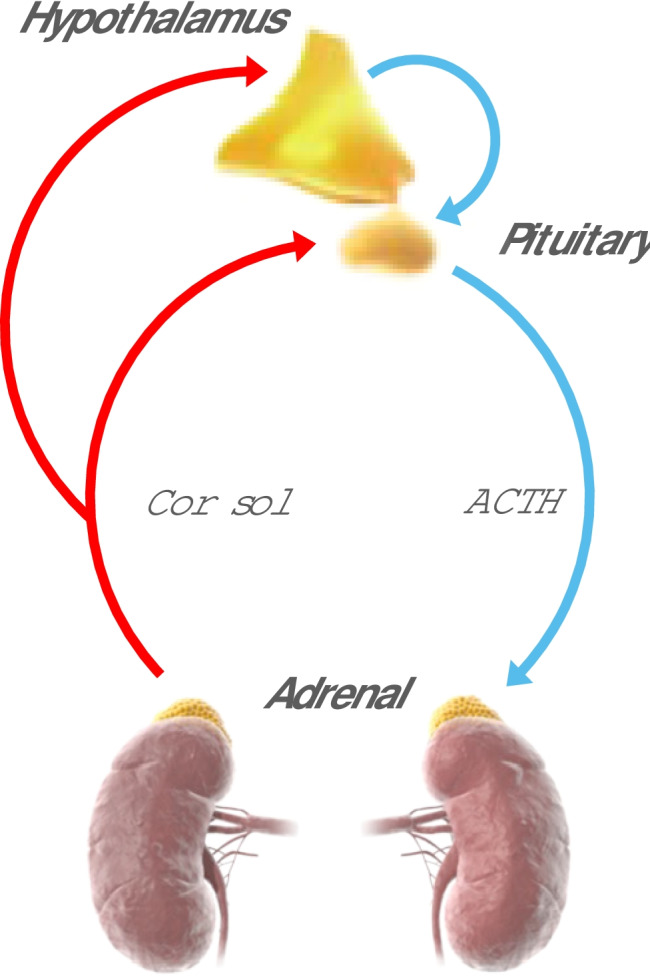
The hypothalamic–pituitary–adrenal (HPA) axis: blue arrows indicate positive cortisol production drive, and the red arrows indicate negative feedback

Cortisol is regulated by adrenocorticotrophin hormone (ACTH) secreted from the pituitary, and there is a classic endocrine negative feedback loop with cortisol feeding back at the hypothalamus and pituitary to inhibit ACTH release (Fig. 2). A combination of the clock drive and negative feedback results in the cortisol circadian rhythm, with cortisol levels rising in the early hours of the morning, peaking around the time of waking and declining through the day to low levels in the evening [8]. Serum cortisol is 80% bound to cortisol-binding globulin (CBG) and 10% to albumin, and approximately, 10% is free cortisol (unbound fraction), with the latter providing biological activity [9]. When cortisol concentration exceeds ~ 550 nmol/L, CBG saturates so that the biologically active free cortisol increases. Cortisol clearance is through a variety of enzyme pathways expressed in many tissues. We first examined hydrocortisone PK in patients with adrenal insufficiency and CAH demonstrating that patients have poor disease control on waking with very low cortisol levels and very high ACTH levels which in CAH drives high androgens that cause infertility [10, 11]. We then trialled an intravenous hydrocortisone infusion to simulate the cortisol circadian rhythm and demonstrated that in adrenal insufficiency, Addison’s and CAH, we could improve disease control, providing proof of concept (PoC) for chronotherapy in adrenal insufficiency [11]. Building on the PoC study, we analysed the cortisol circadian rhythm in healthy individuals and compared our data with the published literature to establish the physiological cortisol profile we wished to replicate (Table 1) [6, 12]. In addition, we developed and verified a physiologically based pharmacokinetic (PBPK) model for the endogenous hormone cortisol (hydrocortisone) in healthy adults and children and in adults with adrenal insufficiency using data from an immediate-release formulation of hydrocortisone, Alkindi^®^ (Diurnal Ltd, Cardiff, UK), which we had formulated to provide accurate dosing in children [13]. Based on this work, we hypothesised that we would need a modified-release formulation of hydrocortisone with both delayed- and sustained-release functionality if we were to fully replicate the physiological cortisol diurnal rhythm.

**Table 1:** Pharmacokinetic characteristics of cortisol circadian rhythm in a healthy reference group and previously published data ( adapted from Debono et al., [6])^9^

**Populations**	**Sample mean age (range)**	AUC (h*mcg/dL)	**Peak (mcg/dL)**	**Trough (mcg/dL)**	**24-h mean cortisol (mcg/dL)**	**Time of peak or acrophase (h:min)**	**Time of nadir (h:min)**	**Quiescent phase start (h:min)**	**Quiescent phase end (h:min)**
**Healthy reference population** (24 males; 9 females)	27 (17–57)	160.2 (148.6–171.6)	16.0 (14.6–17.4)	2.5 (1.8–3.2)	6.7 (5.9–7.5)	08:32 (07:59–09:05)	00:18 (23:39–0:58)	19:43 (18:36–20:49)	05:31 (04:45–06:18)
**Published literature** (127 males; 38 females)	32 (19–59)	169.0 (138.5–207.0)	15.0 (11.6–19.0)	2.04 (1.0–3.2)	7.7 (5.7–15.8)	07:49 (06:28–09:01)	00:30 (22:00–02:00)	19:40 (16:30–22:00)	04:11 (03:00–05:30)

## The ebb and flow of developing a modified-release hydrocortisone

At the outset of the formulation development, we were presented with a range of non-proprietary formulation options, based mainly on conventional multi-particulate technologies, as well as proprietary, patented, formulation options that had claimed specificity for circadian drug delivery. These proprietary technologies shared a common drug delivery objective of conferring both delayed- and sustained-release functionality in one dosage form (Table 2). The claimed mechanisms for drug release, whilst not specifically disclosed in the scientific literature, were centred on established principles of time-dependent polymer erosion, pH-triggered polymer dissolution, osmotic pressure activation and/or diffusional transport. Despite the overt multiplicity of formulation options, few had been evaluated in the clinical setting, and even fewer had been directed towards the circadian delivery of endogenous hormones, with the only exception being the Egalet system, which was used to test the sustained-release delivery for hydrocortisone.

**Table 2:** Selected examples of drug delivery system technologies that have been utilised in marketed pharmaceutical products

**Technology**	**Overview**	**Drug/company**
CODAS^®^ (chronotherapeutic oral drug absorption system)	Non-enteric release-controlling polymers applied to drug-loaded beads where the release-controlling polymer is a combination of water-soluble and waster-insoluble polymers resulting in pH-independent drug release	Verapamil (Elan Drug Technologies, Dublin, Republic of Ireland)
OROS^®^	A ‘push–pull’ multi-layer tablet system for poorly water-soluble drugs. The ‘pull’ drug layer contains the poorly soluble drug, suspending and osmotic agents with the ‘push’ layer containing water-soluble swellable polymers	Nifedipine (ALZA Corp, CA, USA)
Geoclock^®^	Press-coated tablets with slow release outer coat and an immediate-release inner core to allow pH-independent timed release of drug	Prednisolone (SkyPharma, London, UK)
Diffucaps^®^	A multi-particulate bead system comprising multiple layers of drug, excipients and release-controlling polymers	Cyclobenzaprine (Eurand, OH, USA)
Pulsys^®^	Multi-component tablet consisting of one immediate-release and two delayed-release components	Amoxicillin (Middlebrook Pharmaceuticals, MD, USA)
Pulsincap™	Non-disintegrating half capsule body sealed at the open end with a hydrogel plug that is covered with a water-soluble cap	Diclofenac sodium (RP Sherer Corporation, MI, USA)

We initially partnered with a company specialising in electrostatic deposition technology (Phoqus Pharmaceuticals Limited, West Malling, UK) to develop a proprietary circadian formulation for hydrocortisone. The electrostatic deposition technology, at that time, was highly differentiated, as it was the only drug delivery technology that could deposit a precise polymer coating on selective surfaces of a tablet formulation to enable the attainment of both delayed- and sustained-release functionality. The only comparable technology to electrostatic deposition was a press-coated tablet (Geoclock^®^, developed by SkyPharma) utilising compression (press) coating to achieve partial tablet coating, which was applied to the circadian delivery of prednisolone (Lodotra^®^). The principal drawback of press-coating was the inherent difficulty in controlling the uniformity and thickness of the compression coating to minimise pharmacokinetic variations, an aspect which was overcome using the electrostatic deposition technology.

The hydrocortisone circadian dosage form developed using electrostatic deposition was a bilayer modified-release tablet encased with a water-insoluble barrier coating (ammonio methacrylate copolymer, type B) to all but one face of the tablet. The bilayer comprised an active layer containing hydrocortisone and rate-controlling polymers: carbomer homopolymer, type A, and ammonia methacrylate copolymer, type B, surrounded by the barrier coat. The adjoining eroding layer contained an enteric polymer: methacrylic acid copolymer, type A, also surrounded by the barrier coat but with an uncoated exposed surface. The eroding layer was designed to confer delayed-release by preventing the ingress of water into the active layer until complete erosion of the eroding layer had occurred. The duration of delayed-release could thus be optimised by altering the thickness of the eroding layer. The subsequent release rate of hydrocortisone from the active layer could also be independently controlled by altering the polymer concentration so as to alter the diffusional transport of hydrocortisone.

Prototype formulations were developed using electrostatic deposition targeting a delayed-release period of around 3–5 h, followed by a sustained-release period of c. 16–20 h, based on Gastroplus^®^ in silico modelling to achieve close mimicry of the circadian pattern of cortisol. Pharmacokinetic evaluation of two early prototype formulations at a dose of 30 mg in healthy volunteers [14] showed excellent mimicry of the early phase of the circadian pattern of cortisol; however, the level of serum hydrocortisone showed gradual but notable decline from c. 08:00 h, limiting coverage beyond mid-morning.

Follow-on formulations were developed with Phoqus, to refine the release of hydrocortisone, principally by extending the delayed-release duration (from 2 to 4 h) and shortening the sustained-release phase (from 16 to 12 h) to achieve improved serum levels of hydrocortisone from mid-morning onwards. Phase I, pharmacokinetic evaluation demonstrated again good mimicry of the early phase of the circadian pattern of cortisol and a slight improvement to the serum levels of hydrocortisone for the morning exposure compared to the original formulations. In phase I, the exposure of hydrocortisone from these formulations was approximately dose proportional, indicating the absence of dose-limiting absorption.

Formulations were further evaluated in a phase IIa study [15], in patients with CAH to compare the steady-state serum concentration profiles and pharmacodynamic responses relative to immediate-release hydrocortisone (Cortef^®^, Pfizer, USA). The phase IIa study confirmed that the formulations could reproduce the overnight cortisol rise and could improve biochemical control of CAH in the morning. The hurdle of achieving improved levels of serum hydrocortisone from mid-morning onwards for once-a-day dosing was not satisfactorily concluded as Phoqus Pharmaceuticals had ceased its operation for financial reasons, with no prospect for continued development of the electrostatic deposition technology.

**Fig. 3:**
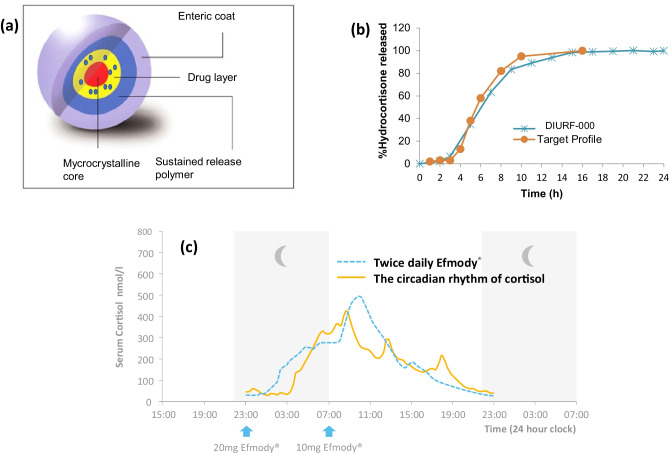
Evolution of Efmody^®^ from concept to performance in patients: **a** micro-particulate formulation; **b** in vitro dissolution of DIURF-000 (in blue) compared to target product profile (orange); **c** phase II cortisol profile in patients with CAH treated with Efmody^®^ (blue) compared to normal cortisol rhythm (orange)

A key learning point from the electrostatic deposition technology experience was that combining a new therapeutic (circadian delivery) concept with a new formulation drug delivery approach greatly increased the risk of failure with two unproven approaches. With that experience, we then turned to the use of a conventional modified-release technology platform, multi-particulates using drug and polymer-layering, to integrate the delayed- and sustained-release features into a revised circadian formulation of hydrocortisone. The key advantage of the multi-particulate technology is that with judicious formulation design, the delayed-release functionality can be controlled independently from the sustained-release functionality, thereby providing scope to optimise the pharmacokinetic profile of hydrocortisone against the circadian pattern of cortisol. The architecture of the base multi-particulate formulation comprised a microcrystalline core layered with hydrocortisone, a sustained-release coat of ammonia methacrylate copolymer, type A and type B, and an outer enteric polymer coat of methacrylic acid-methyl methacrylate copolymer (Fig. 3a).

Although initial in vitro correlation against the selected target product profile was promising (Fig. 3b), pharmacokinetic evaluation in healthy volunteers of a prototype multi-particulate formulation (DIURF-000) with a designed delayed-release duration of 3 to 5 h and a sustained-release duration of 18 h, akin to the original Phoqus Formulation, proved disappointing, marked by variability in drug release onset and generally low serum levels of hydrocortisone caused by a significant shortfall in relative bioavailability (~ 34%). Unlike the Phoqus formulation, which appeared to transit the gastrointestinal (GI) tract slower and more predictably than multi-particulates, the rapid transit of the multi-particulates we presumed had resulted in the majority of the drug payload being delivered to the colon, a region of the GI tract wherein the absorption of hydrocortisone had not been formally studied. A working hypothesis was that hydrocortisone was poorly absorbed in the colon, possibly due to its very low aqueous solubility and limited colonic permeation. In view of the poor bioavailability, three further multi-particulate formulations were developed, all with reduced thickness of the sustained-release coat. Relative bioavailability was improved but remained < 50% compared to immediate-release hydrocortisone, and the shape of the pharmacokinetic profile was similar to that for DIURF-000.

A series of investigative studies were conducted in dogs to determine the optimal ‘absorption window’ for hydrocortisone, enabled by multi-particulate formulations with a significantly shortened sustained-release phase such that the majority of the drug payload would be released in the small intestine. These studies provided definitive proof that the absorption window for hydrocortisone was limited to principally the small intestine and that sustained-release may not be an essential design attribute as hydrocortisone appeared to exhibit dissolution rate-limiting absorption by virtue of its very low aqueous solubility [12].

The multi-particulate formulation was re-designed to significantly reduce the sustained-release component, with one formulation variant being devoid of any sustained-release coating. The re-designed multi-particulate formulations were evaluated in an extended phase I study [12], in healthy volunteers with interim pharmacokinetic readout to permit a revision to the dosing regimen for optimal mimicry of the circadian pattern for cortisol. The results of the pharmacokinetic study correlated remarkably well with the dog study, confirming the selective absorption window for hydrocortisone and verifying critically that sustained-release functionality was indeed not necessary as the intrinsic dissolution rate of hydrocortisone was already sufficiently slow to maintain an absorption input rate that was commensurate with the required target pharmacokinetic profile.

Given the absorption window constraints for hydrocortisone, it was considered not plausible to utilise absorption in the colon to facilitate extended delivery for hydrocortisone, for once-a-day dosing. An alternative approach was adopted using twice-daily dosing via a ‘toothbrush’ regimen to optimise patient compliance, i.e. dosing at night before bedtime and in the morning on waking, to provide full 24-h coverage of hydrocortisone following the circadian pattern of cortisol. This dosing regimen (20 mg at 23:00 h and 10 mg at 07:00 h) was evaluated in the extended pharmacokinetic study in healthy volunteers and was shown to provide consistent physiological levels of hydrocortisone with similar relatively bioavailability to immediate-release hydrocortisone, and the serum concentration was observed to increase linearly with doses between 5 and 30 mg [12]. The formulation used in this study was progressed for clinical development.

## In tempo: clinical development leading to approval

Based on our phase I human volunteer data, we recruited 16 patients with CAH into a 6-month phase II study switching them from their standard glucocorticoid therapy to twice-daily Efmody^®^ and measuring not only pharmacokinetics, the fixed-dose ‘toothbrush’ regimen of 20 mg before bed and 10 mg on waking, but also biochemical disease control at the beginning and end of the trial. In CAH, the enzyme block to cortisol production results in excessive production of a steroid precursor 17OH-progesterone (17OHP) which drives the production of excess adrenal androgens which in turn cause precocious puberty, short stature and infertility in children and adults with CAH. 17OHP control is the biomarker of disease control in CAH. Efmody^®^ resulted in a cortisol profile similar to physiological cortisol levels in healthy volunteers (Fig. 3c) and confirmed that Efmody improved the biochemical control of CAH by reducing the excess morning 17OHP, from 31% of patients in control at the beginning of the study to 94% after 6 months on Efmody^®^ treatment.

**Fig. 4:**
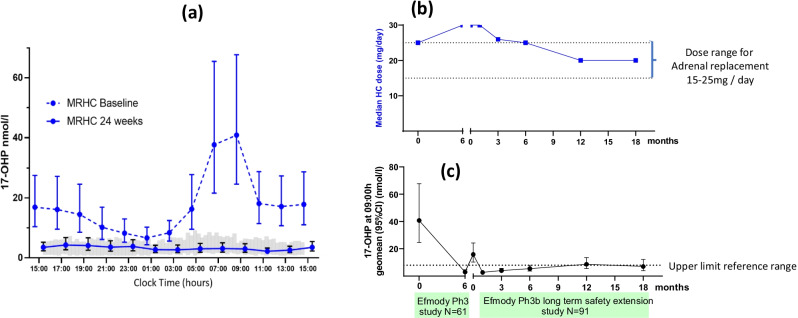
Efmody^®^ provides androgen control in adults with a reduction in steroid dose: **a** pivotal phase III study confirms control of the key androgen 17-OHP throughout the 24-h period and especially in the critical morning period with Efmody^®^ after 24 weeks (grey shading indicates the reference range of 17OHP derived from healthy individuals); **b** in the long-term extension study, optimal disease control was maintained at a reduced daily dose **c** that is recommended for adrenal replacement

Based on these encouraging results from the phase II study and following consultation with the European Medicines Agency (EMA), we then established a multi-centre phase III parallel arm 6-month study of Efmody versus standard therapy followed by an open-label Efmody^®^ extension safety study [16]. The phase III study showed that Efmody^®^ improved disease control of CAH in the morning normalising the 17OHP throughout the day, whereas on standard treatment, 17OHP rose to excessive levels in the morning but was controlled during the day (Fig. 4). The study missed its primary endpoint because this was based on a log-transformed mean of the 24-h data which obscured the beneficial morning suppression of 17OHP. The extension study demonstrated that the improved control of CAH, as measured by 17OHP levels, was maintained on a lower replacement of glucocorticoid, and furthermore, patient-reported clinical benefit was shown with improved fertility with pregnancy in 6 women with CAH and 4 female partners of men with CAH (Fig. 4). The data from the clinical development plan for Efmody^®^ was submitted to the EMA and received marketing authorisation approval from the European Commission on 27 May 2021.

## Lessons learnt

From a formulation design perspective, the use of a delayed- and sustained-release drug delivery approach to achieve circadian delivery remains well supported. However, such an approach will need to be underpinned by rigorous consideration of the absorption window of the compound and fundamentally its ability to be absorbed in the colon. The prediction of colonic absorption in vitro can be challenging, and we have found that the judicious use of an animal model can help to tease out absorption-related issues, as is the case for most modified-release drug delivery development. There remains usually an important trade-off between sustained-release duration and a possible drop-off in bioavailability, which can render once-a-day dosing particularly challenging. We have been able to achieve patient-compliant dosing with the use of a ‘toothbrush’ regimen which overcomes the need for once-a-day dosing for patients with CAH. This drug delivery approach, using conventional multi-particulate technology, delivers physiological levels of hydrocortisone that commensurate with the circadian pattern of cortisol and a bioavailability close to that of the immediate-release formulation. This program shows that it is possible, through application of drug delivery technology, to develop physiological drug replacement products that can treat chronic endocrine diseases to preserve health for life.
